# Management of auricular transcutaneous neuromodulation and electro-acupuncture of the vagus nerve for chronic migraine: a systematic review

**DOI:** 10.3389/fnins.2023.1151892

**Published:** 2023-06-15

**Authors:** David Fernández-Hernando, Cesar Fernández-de-las-Peñas, Juan A. Pareja-Grande, Francisco J. García-Esteo, Juan A. Mesa-Jiménez

**Affiliations:** ^1^Universidad San Pablo-CEU, CEU Universities, Urbanización Montepríncipe, Boadilla del Monte, Spain; ^2^Department of Physical Therapy, Occupational Therapy, Physical Medicine and Rehabilitation, Universidad Rey Juan Carlos, Alcorcón, Spain; ^3^Neurology, Fundación Hospital Alcorcon, Alcorcon, Madrid, Spain; ^4^Facultad de Medicina, Universidad San Pablo-CEU, Madrid, Spain; ^5^Research Laboratory INCRAFT (Interdisciplinary Craniofacial Pain Therapy), Madrid, Spain

**Keywords:** systematic review, migraine, non-invasive neuromodulation, vagus nerve, electro-acupuncture

## Abstract

**Background:**

Migraine is a type of primary headache that is accompanied by symptoms such as nausea, vomiting, or sensitivity to light and sound.

**Objective:**

The aim of this study was to conduct a systematic review on the effectiveness of non-invasive neuromodulation, auricular transcutaneous vagus nerve stimulation (at-VNS), and electro-ear acupuncture of the vagus nerve in patients with migraine headaches.

**Methods:**

Six databases were searched from inception to 15 June 2022 for clinical trials, in which at least one group received any form of non-invasive neuromodulation of the vagus nerve for managing migraine with outcomes collected on pain intensity and related disability. Data, including participants, interventions, blinding strategy, outcomes, and results, were extracted by two reviewers. The methodological quality was assessed with the PEDro scale, ROB, and Oxford scale.

**Results:**

The search identified 1,117 publications with nine trials eligible for inclusion in the review. The methodological quality scores ranged from 6 to 8 (mean: 7.3, SD: 0.8) points. Low-quality evidence suggests some positive clinical effects for the treatment of chronic migraine with 1 Hz with at-VNS and ear-electro-acupuncture compared with the control group at post-treatment. Some of the studies provided evidence of the relationship between chronic migraine and a possible positive effect as a treatment with at-VNS and the neurophysiological effects using fMRI. Six of the studies provided evidence using fMRI of the relationship between chronic migraine and a possible positive effect as a treatment with at-VNS and the neurophysiological effects. Regarding all included studies, the level of evidence with the Oxford scale was level 1 (11.17%), six studies were graded as level 2 (66.66%), and two studies were graded as level 3 (22.2%). With the PEDro score, five studies got a low methodological score < 5 and only four got a score superior to 5, being highly methodological quality studies. For ROB, most of the studies were high risk and only a few of them received a low risk of bias. The pain intensity, migraine attacks, frequency, and duration were measured by three studies with positive results at post-treatment. And only 7% reported adverse events using at-VNS. All studies reported results at a post-treatment period in their respective main outcomes. And all studies with fMRI provided strong evidence of the relationship between the Locus Coeruleus, Frontal Cortex, and other superior brain areas with the auricular branch of the Vagus nerve with at-VNS.

**Conclusion:**

Some positive effects regarding the effect of non-invasive neuromodulation, auricular transcutaneous vagus nerve stimulation (at-VNS), and electro-ear acupuncture of the vagus nerve on migraine is reported in the current literature, but there are not enough data to obtain strong conclusions.

**Systematic review registration:**

This systematic review was registered in the PROSPERO database (registration number: CRD42021265126).

## Introduction

Migraine is a type of primary headache that causes disability, reduces quality of life, and affects more than 1 billion people worldwide each year. The worldwide prevalence of migraine is around 11.6% (Stovner and Andree, 2010; Vetvik and MacGregor, 2017; Woldeamanuel and Cowan, 2017), with the majority of patients being under 50 years of age, and it affects more females than males (Stovner and Andree, 2010). Globally, migraines are the second-most frequent cause of disability, responsible for 16.3% of neurological symptoms and a significant impact on daily living activities (Amiri et al., 2022).

While the actual etiology of migraines is currently unknown, one hypothesis that may explain their development is the convergence of cervical and trigeminal afferents in the trigeminal–cervical nucleus (GBD 2016 Headache Collaborators, 2018; Huang et al., 2020). There are indicators of neurogenic inflammation associated with this primary headache and a hypersensitive immune system related to central sensitization (Grassini and Nordin, 2017). There is evidence for the association of headache pain with alterations in the brainstem nuclei and cortical regions, while migraine headaches involve increased sensory processing within the peripheral and central trigemino-vascular pathways and the relationship between them (Song et al., 2022).

It is likely that the main cause of migraine is the imbalance between excitatory and inhibitory cortical–subcortical neurotransmission. This abnormal interaction between neurons has been termed the phenomenon of “Cortical Diffuse Depression (CSD)”, involving the vascular system and the release of inflammatory mediators modulating neuronal activity (de Tommaso et al., 2021).

Recently, the existence of a trigeminovagal complex has been reported as the basis for connections between the trigeminal and vagus systems, as well as its possible connection with CSD, as a possible relationship of this cranial pair with primary headaches. These findings have been confirmed with functional MRI in humans, and vagus nerve stimulation can modulate the trigeminal autonomic reflex through a complex network which includes the hypothalamus, the trigeminal spinal nuclei, the left pontine nucleus, and the parahippocampal gyrus (Henssen et al., 2019a; Möller et al., 2020).

Traditionally, abortive and prophylactic medications are first-line treatments for migraine therapy, with most migraineurs treating their headaches at the onset of symptoms. The principal treatment options are the 5-HT_1F_ receptor, non-steroidal anti-inflammatories (NSAIDs), Calcitonin gene-related peptide (CGRP), and gepants, but these types of treatments may cause side effects with long-term use, such as gastric ulcer disease and chronic kidney diseases, which are not well tolerated and may increase the risk of medication overuse, headaches, allodynia, and dependence (Blech and Starling, 2020). The limitations of current pharmacological therapies have highlighted the need to explore alternative or integrative treatments for migraine.

One potential non-pharmacological approach to the treatment of migraine patients is auricular transcutaneous vagus nerve stimulation (at-VNS), which is commonly used in clinical practice for treating migraine, cluster headache, depression, epilepsy, and other disorders, such as atrial fibrillation, prosocial behavior, associative memory, schizophrenia, or pain (Usichenko et al., 2017; Badran et al., 2018; Kaniusas et al., 2019; Yap et al., 2020). At-VNS is a non-invasive and inexpensive therapy that involves stimulating the auricular branch of the vagus nerve (ABVN) at the outer parts of the ear, conferring autonomic benefits (Badran et al., 2018). One of the main differences between VNS and at-VNS is that patients do not require general anesthesia for its implantation, thus making at-VNS safer than VNS (Usichenko et al., 2017; Badran et al., 2018; Kaniusas et al., 2019; Blech and Starling, 2020; Yap et al., 2020).

The former method is also more expensive and riskier than at-VNS, with costs ranging from USD 30,000 to USD 50,000 (Usichenko et al., 2017; Badran et al., 2018; Blech and Starling, 2020; Yap et al., 2020). Magnetic resonance imaging (MRI) has recently shown anatomical paths for performing at-VNS, with examples including through the neck and auricula, and acupuncture points in the ear, as they are a connection between the nervous system and the external parts of the body (Rong et al., 2012; Badran et al., 2018; Kaniusas et al., 2019; Zhang et al., 2019). Although the physiological effects of at-VNS on the brain have not yet been fully elucidated, and studies are not homogeneous in their results due to the high risk of bias and unclear parameters, stimulation intensity, pulse width, waveform, or frequency and acupuncture points selection, there still exists a path between the auricular and neck branches to the superior brain areas related to at-VNS (Badran et al., 2018; Zhang et al., 2019). On the other hand, at-VNS and electro-acupuncture have analgesic effects with low frequency in various pain models in humans and rodents. Additionally, the common parameters of frequency are between 1 and 20 Hz, and have a high relevance as a treatment for migraineurs using at-VNS (Ellrich, 2006; Feng et al., 2022; Sacca et al., 2022). Other studies obtained findings about a neurophysiological effect between at-VNS and the auricular branch of the vagus nerve related to superior brain areas with the parameters 500 μs ad 25 Hz, which connects cortical effects that may provide findings for future research. However, it is necessary to understand the possible changes with the proper stimulation. In recent years, findings about the relationship between parasympathetic innervation of the vagus nerve and superior areas of the brainstem, such as the locus coeruleus, nucleus tractus solitarious, and trigeminal spinal tract, have been identified. This provides strong evidence regarding the main role of at-VNS for the management of the pathophysiology of migraine (Simon and Blake, 2017; Henssen et al., 2019b; Zhang et al., 2019). Findings have been made regarding four core areas, namely autonomic nervous system function, inhibition of cortical spreading depression (CSD), neurotransmitter regulation, and nociceptive modulation, as a complex mechanism for the treatment of migraines (Silberstein, 2020).

The growing literature and findings encourage the use of at-VNS as an effective technique for the treatment of migraine, and may have the same mechanisms as auricular acupuncture, applied in the auricular region of the vagus nerve (Usichenko et al., 2017; Hamer and Bauer, 2019).

Currently, there are no systematic reviews of non-invasive auricular vagus nerve electrical neuromodulation (at-VNS) and auricular electro-acupuncture in the treatment of chronic migraine, so the purpose of the following systematic review is to assess the efficacy and quality of studies of at-VNS and electro-acupuncture in the auricular region of the vagus nerve for the treatment of chronic migraine (He et al., 2012; Tobaldini et al., 2019).

## Methods

A systematic review following the PRISMA statement (Page et al., 2021) was conducted. This systematic review was registered in the PROSPERO database (registration number: CRD42021265126).

### Search strategy

The electronic databases CINALH, MEDLINE, PUBMED, PEDro, and EMBASE were searched by a reviewer up to June of 2022. Search strategies for each database were based on PICO, and eligible studies were included with a population suffering from migraine (acute or chronic) who had been treated with n-NVS, auricular Transcutaneous Vagus Nerve Stimulation (at-VNS), t-VNS, ear-electro-acupuncture, or non-invasive treatments compared with a control group, sham, or other intervention. The studies which were included were RCTs, controlled clinical trials, clinical trials, and pilot studies. The used MeSH terms and free terms included:

“Vagus Nerve”, “Auricular Vagus Nerve Stimulation”, “Transcutaneous Vagus Nerve Stimulation”, “electro-acupuncture”, “ear-electro-acupuncture”, “migraine”, and “headache”. The concepts were combined with the “AND” or “OR” operators. Additionally, the combination of Mesh terms was (“auricular vagus nerve stimulation” OR “auricular t-VNS” OR “non-invasive vagus nerve stimulation” OR “electro-acupuncture” OR “ear-electro-acupuncture”) AND (“migraine” OR “migraine” OR “headache” OR “headache”).

### PICO question

This systematic review was conducted to answer the following clinical question: “Is non-invasive neuromodulation effective for the management of chronic migraine?”

Population: Adults with Chronic migraine older than 18 years of age.

Intervention: Application of auricular transcutaneous vagus nerve stimulation technique and ear electro-acupuncture.

Comparator: Acceptable comparators were any type of placebo (e.g., turning-off device), sham, or no intervention of pain, duration of symptoms, and/or adverse events, healthy subjects or those with another type of pathology.

Outcomes: The primary outcomes measured were intensity and frequency of migraine attacks.

The studies have to fully answer all these questions to be part of the acceptable or suitable studies to be included in the systematic review.

### Study selection

This systematic review was limited to randomized controlled trials, clinical trials, and controlled trials. Studies were excluded if they were a series of cases, case reports, retrospective and prospective cohorts, notes to editors, chapters of books, trials on animals, or articles with only manual acupuncture as a treatment group. The intervention groups were at-VNS, n-VNS, or ear-electro-acupuncture, and the control group should receive alternative interventions to be able to compare effects (e.g., different places for intervention treatment, turn-off devices, and different treatments) and at least one outcome should be reported at the conclusion of the intervention in chronic migraine.

### Data extraction and quality assessment

Data extraction was performed by two authors and the data were compiled into a standardized data extraction form in an Excel spreadsheet. Data included simple size, diagnosis, inclusion/exclusion criteria, duration of symptoms, intervention type (location, technique, and duration), main outcomes, time to outcome, and adverse events. In case of discrepancy between authors, an agreement should be achieved. If no achievement is reached, a third author should be in charge of reaching a consensus.

The methodological quality of the trials was evaluated with the PEDro (Physiotherapy Evidence Database) scale and the ROB-2 Cochrane tool independently by two authors. The RoB-2 tool includes the following items: selection bias (randomization sequence generation, allocation concealment), performance bias (blinding participants, blinding therapists), detection bias (blinding outcome assessor), attrition bias (incomplete outcome data), reporting bias (source of funding bias/selecting outcome reporting), and other bias (sample size). Each item was classified as low risk, high risk, or unclear according to the Cochrane Collaboration tool (Higgins et al., 2011). In all cases, the answer “Yes” indicates a low risk of bias, and the answer “No” indicates high risk of bias. If insufficient details are reported of what occurred during the trial, or the entry was not relevant to the study (particularly for assessing blinding and incomplete outcome data, when the outcome being assessed by the entry has not been measured in the study), the answer was “unclear” risk of bias.

The PEDro scale is based on 11 criteria, of which 10 contribute to the score, representing methodological quality. The first item is not included (but should always be fulfilled) in the score, as it relates to the external validity of the study. The PEDro scale has been shown to have fair-to-good inter-rater reliability (ICC 0.55, 95% CI 0.41–0.72). The PEDro score assessed the following items: random allocation, concealed allocation, between-groups similarity at baseline, participant blinding, therapist blinding, assessor blinding, dropout, intention-to-treat statistical analysis, between-group statistical comparison, point measures, and variability data (Luo et al., 2020). A PEDro score equal to or >5 out of 10 points determined a high-methodological-quality trial. Higher scores indicated higher methodological quality (total score from 0 to 10) (Maher et al., 2003). Trials with a PEDro score ≥5 points were considered to be of high quality. Disagreements between reviewers were resolved by a third reviewer.

Quality of evidence was rated according to the Oxford Center for Evidence-Based Medicine. Levels of Evidence evaluated included: level 1, randomized trials or systematic reviews of randomized trials; level 2, randomized trial or (exceptionally) observational study with dramatic effect; level 3, non-randomized controlled cohort/follow-up study; level 4, case series, case–control study, or historically controlled studies; and level 5, mechanism-based reasoning. Level 1 represented the likely strongest evidence, and level 5 represented the likely weakest evidence (Marx et al., 2015).

## Results

### Study selection and characteristics

**Figure 1:**
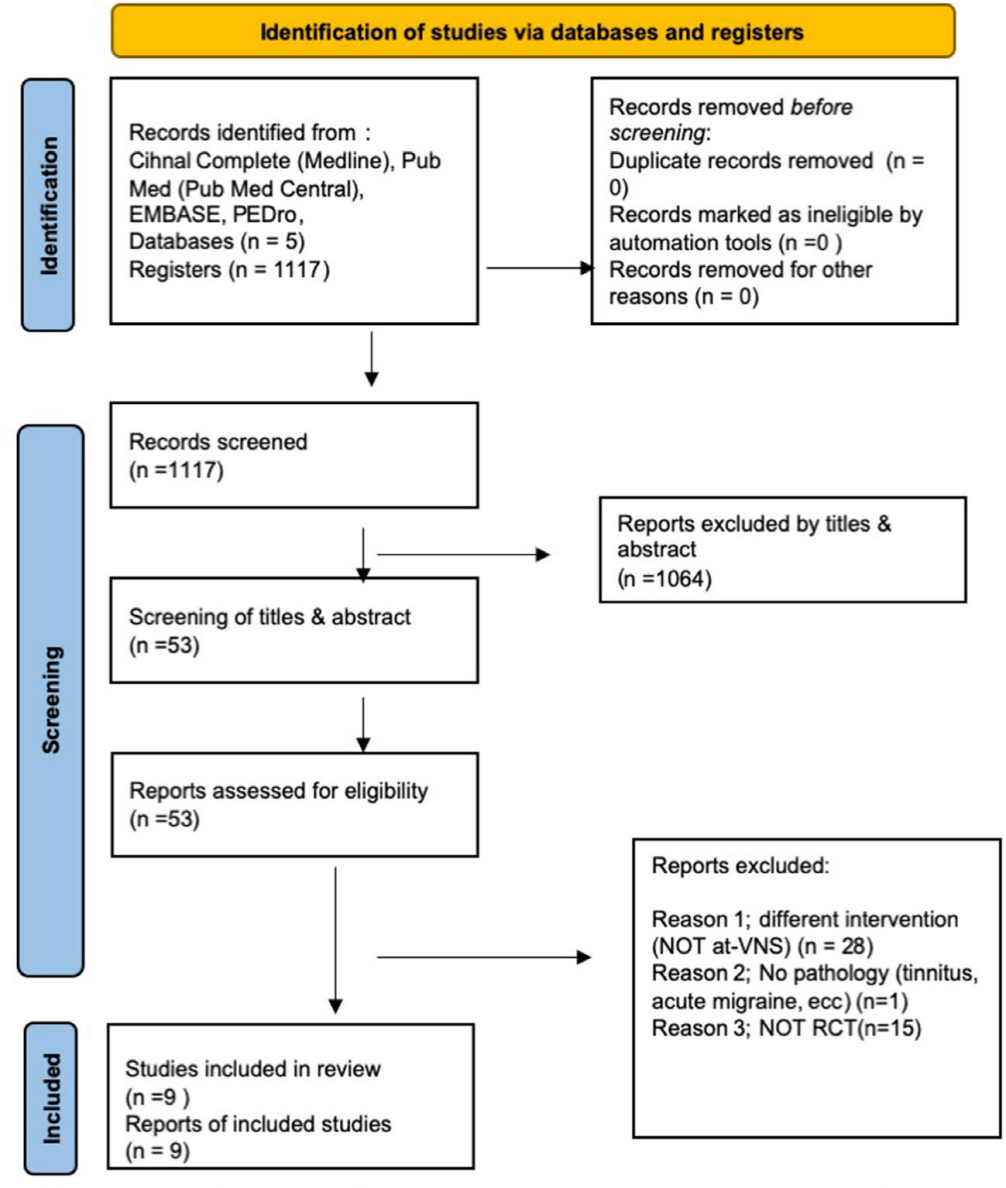
Flow diagram of study selection. RCT, randomized controlled trial.

The data search yielded a total of 1,117 articles, including duplicates. We excluded many articles based on title and abstract (*n* = 1064), and eleven (*n* = 11) because of repetition of the potential eligible articles. Forty-two (*n* = 42) articles were included for abstract/full-text review, of which thirty-three were excluded because of other pathologies or areas of application or another type of pathology such as cluster headache, headache, acute migraine, tinnitus, cervical area of treatment (*n* = 33). Finally, only nine were included in the systematic review (Vijayalakshmi et al., 2014; Yang et al., 2014; Straube et al., 2015; Zhang et al., 2019, 2021; Luo et al., 2020; Cao et al., 2021; Sacca et al., 2022; Wei et al., 2022). Figure 1 shows the flow diagram. The strategy of each database is represented in Table 1.

**Table 1:** Search strategy.

**Database**	**Search number**	**Search strategy**	**Number**
PubMed	#1	(“auricular vagus nerve stimulation”[Mesh])	439
	#2	(“auricular t-VNS” OR “noninvasive vagus nerve stimulation” OR “electro-acupuncture” OR “ear-electro-acupuncture”)	992
	#3	#1 OR #2	3
	#4	“Migraine”[MeSH]	46,525
	#5	OR headache	117,159
	#6	#4 OR #5	26,314
	#7	#3 AND #6	29
CINAHL (MEDLINE)	#1	(“auricular vagus nerve stimulation” OR “auricular t-VNS” OR “non invasive vagus nerve stimulation” OR “electro-acupuncture” OR “ear-electro-acupuncture”) AND (“migraine” OR “headache”)	21
EMBASE	#1	(“auricular vagus nerve stimulation” [Mesh])	676
	#2	(“auricular t-VNS” OR “non invasive vagus nerve stimulation” OR “electro-acupuncture” OR “ear-electro-acupuncture”)	9,655
	#3	#1 OR #2	9,941
	#4	“Migraine” ([Mesh])	87,466
	#5	OR Headache	345,227
	#6	#4 OR #5	381.197
	#7	#3 AND #6	741
PEDro	#1	Migraine ([Mesh])	325
	#2	Non invasive vagus nerve stimulation ([Mesh])	4
	#3	Noninvasive vagus nerve stimulation AND migraine	2
	#4	#1 AND #2 AND #3	2

Table 2 summarizes the studies included in the review. The total sample size consisted of 333 participants (mean age: 34.78, SD: 4.95, 41.74% women). The duration of migraine-associated symptoms was 14.61 (SD: 7.17 years) and the frequency of attacks per month was 10.8 (SD: 5.5).

**Table 2:** Summary of all included studies.

**References**	**Intervention(s)**	**Sample size**	**Age (years)**	**Intervention duration (sessions/weeks)**	**Area of treatment**	**Comparison and outcome measure**	**Results**	**Adverse effects**	**Duration migraine**	Evidence level^*^
**Migraine**										
Cao et al. (2021), China	G1; taVNS = NR; 20-Hz. G2; taVNS = NR 1 Hz;	*N =* 24	31.33 ± 1.55	taVNS stimulation lasted about 8 min. Unique session	Left concha (cymba and cavum)	VAS, MSQ, SDS, SAS, number migraines fMRI G1 vs. G2	G2; taVNS (1Hz) improved FC in PAG, bilateral MCC, right precuneus/posterior cingulate cortex, left MFG, left cuneus, left insula, ACC. Number migraines better with 1Hz ta-VNS. NS VAS, MSQ, SDS, SAS	None	8.68 ± 1.47 y.	2
Wei et al. (2022), China	G1; EA G2; healthy people	*N =* 30	32.285 ± 6.41	2Hz, 1 mA for 8 min until the end of fMRI scan	GB 8	HRSD-17 & HRSA-14	G1 could inprove (GB8) FC between the right insula subregions and parietal lobe, namely, the right dAI and right postcentral gyrus, and the right PI and left precuneus	None	16.20 ± 8.19	3
Sacca et al. (2022), USA	G1; taVNS =; 1-Hz. G2; taVNS = 20 Hz	*N =* 20	31.33 ± 1.55 y	taVNS stimulation 1Hz, 8 minutes, 4 mA, 2 session with fMRI scan	Left concha (cymba and cavum)	Migraine duration, number of migraine attacks, VAS, Migraine Specific Quality of Life Questionnaire, fMRI	G1 Improved NTS/LC–occipital cortex sFC and a decrease of NTS-thalamus sFC, greater LC precuneus and LC–inferior parietal cortex sFC than G2. G1 decreased NTS–postcentral gyrus dFC than G2. G2 compared with baseline increase of the LC–anterior cingulate cortex (ACC) sFC.	None		2
Zhang et al. (2021), China	G1; taVNS = 33, 1Hz, 0.2ms G2; Sham group = 26	*N =* 70	NR	30 min of 12 treatment sessions in total during the 4 week treatment	Left cymba concha	VAS MSQ Migraine attack times SAS fMRI G1 vs. G2	G1 SF number of migraine days (p = 0.024) G1 SF (p = 0.008), migraine attack times (p = 0.015) NS MSQ, SAS, SDS fMRI G1; FC, occipital' thalamic seed and the bilateral PoG reduction of the migraine days (p = 0.016) G2; NS	None	4.0 (1.9) days 4.0 (3.2) days	1
Luo et al. (2020), China	G1; taVNS 1Hz, 0.2ms. G2s; tVNS Sham.	*N =* 27	29.85 ± 8.09	Unique session of MRI scan, total of six 20-min fMRI runs and 8 min ta-VNS	CO11 and CO14, left cymba concha	VAS, MSQ, SAS, SDS, fMRI G1 vs. G2	G1; FC Improved, left amygdala, left MFG, right SMA, left dorsolateral superior frontal gyrus, bilateral paracentral lobules, bilateral postcingulum gyrus, and right frontal superior medial gyrus. Left FC and right SMA in frequency/time in migraine in 4 weeks.	None	NR; NR	2
Zhang et al. (2021), China	G1; ta-VNS, 1Hz, 0.2ms. G2; Sham ta-VNS	*N =* 26	32.50 ± 7.57	MRI session of 30 minutes	Left cymba concha	VAS, MSQ, SDS, SAS, fMRI G1 vs. G2	G1 greater deactivation at the bilateral LC. rsFC the right temporoparietal junction and left secondary somatosensory cortex (S2) SF increased vs. G2	None	7.15 ± 2.87 Y. 3.23 ± 1.58 mo	3
Straube et al. (2015), Germany	G1; taVNS 25 Hz, 250 μs, cycle: 30s on, 30 s off G2; ta-VNS 1 Hz; 250 μs, cycle: 30s on, 30 s off	*N =* 46 *N =* 24 *N =* 22	41,55 ± 11,95	4 h per day during 12 weeks	Concha of the outer ear	Pain Imtensity (NRS), MIDAS, HIT-6, BDI G1 vs. G2	G2 SF headache days, (p = 0.035). HIT-6 & MIDAS SF G1 & G2	Only 3 of 46 patients (7%) dropped out due to side effects of t-VNS.	20.4 ± 12.1 years 27.1 ± 13.0 years	2
Yang et al. (2014), China	G1; AG; G2; SAG; G3; MG	*N =* 30	33.28 ± 8.03	30 min of unique session 30 min Sham acupucnture 40 min rest	TE8, TE19, GB33	VAS PET-CT	G1 & G2 VAS SF (P < 0.05) MG NS(P = 0.047) AG vs. MG middle frontal gyrus, postcentral gyrus, the precuneus, parahippocampus, cerebellum and middle cingulate cortex (MCC), and decreased in the left hemisphere of (MTC) SAG vs. MG posterior cingulate cortex (PCC), insula, inferior temporal gyrus, MTC, superior temporal gyrus, postcentral gyrus, fusiform gyrus, inferior parietal lobe, superior parietal lobe, supramarginal gyrus, middle occipital lobe, angular and precuneus cerebellum, parahippocampus	None	NR NR	2
Vijayalakshmi et al. (2014), India	G1; Electro acupuncture; G2; Drug therapy	*N =* 60 *N =* 30 each group	NR	10 sessions for 30 days (0.5 mA; 10-20 Hz) flunarizine 20 mg OD and tab. paracetamol 500 mg SOS for 30 days	DU 20, P.6, St.36, GB.41, GB.14, EM, LI.4, LI.10, ST.44, Ear points: Ear shenmen and Ear stomach (16)	MIDAS WHO QOL BREF	G1 SF (P = 0.005–0.000) in all outcomes.	None	NR NR	2

VAS, visual analog scale; MSQ, Migraine Specific Quality of Life; SDS, Self-rating Depression Scale; SAS, Self-rating Anxiety Scale; NVS, transcutaneous auricular-Nerve Vagus stimulation; fMRI, functional Magnetic Resonance Image; AG, Electro-acupuncture group; SAG, Sham Acupunture Group; MG, Migraineur Wait-List control Group; WHO QOL BREF.

* Levels of Evidence based on the Quality Rating Scheme for Studies and Other Evidence modified from the Oxford Centre for Evidence-Based Medicine for rating of individual studies; available online at https://www.cebm.net/2016/05/ocebm-levels-of-evidence/.RCT.

NR, Not reported; LC, locus coeruleus; rsFC, Resting state functional connectivity; SF, Significance; EA, Electro-acupuncture; PCC, Posterior cingulate cortex; MTC, Middle Temporal Cortex; SMA, supplementary motor area; MFG, middle frontal gyrus; MFC, Middle Frontal Cortex.

### Outcomes

We extracted the following outcomes: the Visual Analogic Scale (VAS) (Vijayalakshmi et al., 2014; Zhang et al., 2019, 2021; Luo et al., 2020; Cao et al., 2021); the frequency and duration of migraine attacks (Yang et al., 2014; Straube et al., 2015; Zhang et al., 2019, 2021; Luo et al., 2020; Cao et al., 2021); Quality-of-life Questionnaire (QoL) (Vijayalakshmi et al., 2014; Cao et al., 2021); Self-rating Depression Scale (SDS); Self-rating Anxiety Scale (SAS); Migraine-Specific Quality of Life (MSQ) (Yang et al., 2014; Straube et al., 2015; Zhang et al., 2019, 2021; Luo et al., 2020; Cao et al., 2021); Migraine Disability Assessment (MIDAS) (Vijayalakshmi et al., 2014; Straube et al., 2015) and the Headache Impact Test (HIT-6) (Straube et al., 2015); WHO Quality-of-Life BREF (Biomedical Research and Education Foundation) (Vijayalakshmi et al., 2014); HRSD-17 and HRSA-14 (Wei et al., 2022); Migraine-Specific Quality-of-Life Questionnaire; and fMRI scan (Sacca et al., 2022).

There were different post-treatment follow-up periods for respective outcomes. Zhang et al. (2019) assessed the pain intensity and frequency of migraine attacks using MSQ, SDS, and SAS at post-treatment after just a single session. Cao et al. (2021) performed all measurements, namely migraine duration, migraine attacks, average of pain intensity with VAS, and MSQ at post-treatment for all outcomes. Straube et al. (2015) assessed chronic migraine-related disability with the HIT-6 and MIDAS at 14 days, 28 days, and 56 days after starting the treatment, as well as at the end of the study 12 weeks afterwards. Luo et al. (2020) took all measurements at post-treatment (one week). Additionally, Vijayalakshmi et al. (2014) and Yang et al. (2014) took all measurements at post-treatment only.

### Interventions

For the intervention group, all studies investigated at-VNS or ear electro-acupuncture. The at-VNS devices were placed for most of the studies in the left side of the cymba concha (Straube et al., 2015; Zhang et al., 2019; Cao et al., 2021), left cymba concha, and at the concha of the outer ear (Zhang et al., 2021; Sacca et al., 2022), and there were three studies that chose acupuncture points, namely TE 19 (Yang et al., 2014), ST16 (Vijayalakshmi et al., 2014) and CO11, CO14 (Luo et al., 2020), and GB8 (Wei et al., 2022). Accordingly, the area of stimulation was the auricular branch of the vagal nerve.

The parameters of electrical stimulation were poorly described, with a pulse width ranging from 150 to 200 ms. The intensity of the current was the strongest stimulation tolerable for the subjects and a frequency ranging from 1 to 20–25 Hz. The number and time of sessions were also different among studies, most of which included short periods of time between application during the migraine attack, usually 8 min (Straube et al., 2015; Cao et al., 2021; Sacca et al., 2022), whereas one study specifically applied 12 sessions of 30 min each (Luo et al., 2020), a daily application of 4 h during 12 weeks of the study, and 1 during the MRI scan session of 30 min to check the at-VNS effectivity (Cao et al., 2021). Wei et al. (2022) used a device with 2Hz and 1 mA with a continuous wave for 8 min during the fMRI scan at the GB8 acupuncture point. Sacca et al. (2022) used a device with 1 Hz in 8 min and 4 mA in the left concha (cymba and cavum) during 2 fMRI.

The usual sham at-VNS in most of the studies was the placement of the electrode in another anatomical body area, e.g., left tail of the helix (Zhang et al., 2019; Cao et al., 2021), or using a sham device without stimulation (Straube et al., 2015), at-VNS with different parameters (Straube et al., 2015; Zhang et al., 2019; Cao et al., 2021; Sacca et al., 2022), or other acupuncture points further from the ear (Vijayalakshmi et al., 2014; Yang et al., 2014), with non-vagal fibers (Luo et al., 2020) or healthy subjects (Wei et al., 2022).

### Methodological quality, risk of bias, and quality of evidence

**Figure 2:**
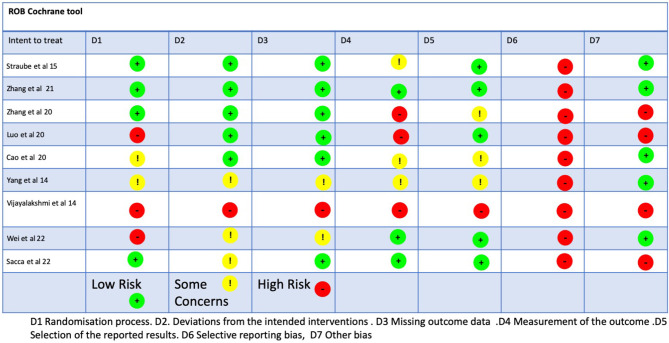
ROB2 tool.

**Figure 3:**
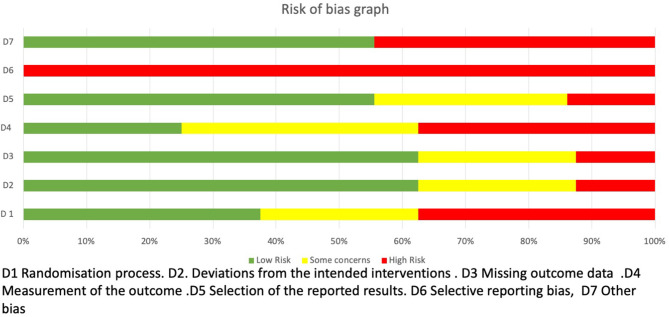
Graph of the ROB2.

The methodological quality scores ranged from 6 to 8 (mean: 7.3, SD: 0.8) out of a maximum of 10 points (Table 3). Four studies (Straube et al., 2015; Luo et al., 2020; Cao et al., 2021; Zhang et al., 2021) obtained a high methodological quality (≥5 points) and five obtained a low methodological quality (Vijayalakshmi et al., 2014; Yang et al., 2014; Sacca et al., 2022; Wei et al., 2022). The ROB-2 Cochrane tool identified a low risk of bias for two studies (Zhang et al., 2021), one with some concerns (Cao et al., 2021), and four studies presented a high risk of bias (Vijayalakshmi et al., 2014; Yang et al., 2014; Straube et al., 2015; Luo et al., 2020; Wei et al., 2022) (Figure 2). The risks of bias identified were the following: random sequence generation (D1): 44.4% low risk, 22.2% some concerns, and 33.3% high risk; allocation concealment (D2): 55.5% low risk, 33.3% some concerns, and 11.1% for high risk; blinding of participants and researchers (D3): 66.6% for low risk, 22.2% for some concerns, and 11.1% for high risk; blinding of outcome assessment (D4): 33.3% low risk, 33.3% for some concerns and for high risk; incomplete outcome data (D5): 55.5% low risk, 33.3% some concerns, and 11.1% high risk, (D6): any of them reported the sample size calculation with a high risk of bias (100%), (D7). Some of the studies reported the clinical trial registration number, giving them a low risk of bias (55,5%) but some did not report the prospective registration, which is now a requirement of the revised Declaration of Helsinki, giving them a high risk of bias (44.4%) (Figure 3). Based on the Oxford grading of evidence, one study was graded as level 1 (11.17%) (Zhang et al., 2021), six studies were graded as level 2 (66.66%) (Vijayalakshmi et al., 2014; Yang et al., 2014; Straube et al., 2015; Luo et al., 2020; Cao et al., 2021; Sacca et al., 2022), and two studies were graded as level 3 (22.2%) (Zhang et al., 2019; Wei et al., 2022). Study characteristics are detailed in Table 2.

**Table 3:** Score of randomized clinical trials with PEDro scale.

	**1**	**2**	**3**	**4**	**5**	**6**	**7**	**8**	**9**	**10**	**Total**
**Migraine**											
Wei et al. (2022)	N	N	Y	N	N	N	N	N	Y	Y	3/10
Sacca et al. (2022)	Y	N	Y	N	N	N	N	N	Y	Y	4/10
Cao et al. (2021)	Y	N	Y	Y	N	N	Y	Y	Y	Y	7/10
Zhang et al. (2021)	Y	N	Y	Y	N	Y	N	Y	Y	Y	7/10
Luo et al. (2020)	Y	N	Y	Y	N	N	Y	N	Y	Y	6/10
Zhang et al. (2019)	N	Y	Y	Y	N	N	N	Y	Y	N	5/10
Straube et al. (2015)	Y	N	Y	Y	N	N	N	Y	Y	Y	6/10
Yang et al. (2014)	Y	N	N	Y	N	N	N	N	N	Y	3/10
Vijayalakshmi et al. (2014)	Y	N	N	N	N	N	N	N	N	N	2/10

1: Random Allocation of Participants; 2: Concealed Allocation; 3: Similarity Between Groups at Baseline; 4: Participant Blinding; 5: Therapist Blinding; 6: Assessor Blinding; 7: Fewer than 15% Dropouts; 8: Intention- to-Treat Analysis; 9: Between- Group Statistical Comparisons; 10: Point Measures and Variability Data.

Y, Yes, If the criterion is satisfied.

N, No, If the criterion is not satisfied.

### Summary of results

We found low-quality evidence showing some positive effects on the intensity and frequency of migraine attacks. There was one protocol that compared two at-VNS groups (pulse width: 250 μs; frequency: 1 or 25 Hz; duty cycle: 30 s on/30 s off, 4 hour/day for 12 weeks) and found positive effects in the reduction of days with migraine and also HIT-6 and MIDAS after 12 weeks with at-VNS at 1 Hz, compared with the other group with 25Hz (Straube et al., 2015). Meanwhile, a completely different protocol employed two groups with different frequencies and time of at-VNS application, compared 20 Hz and 1 Hz of frequency of at-VNS with a width of 0.2 ms for 8min, and observed that at-VNS with 1 Hz might be beneficial for reducing chronic migraine pain as an alternative and safe treatment. Additionally, pre-treatment and post-treatment (4 week) VAS, MSQ, SDS, and SAS were assessed. Positive results were only seen in VAS and number of migraine attacks (Cao et al., 2021). On the other hand, three groups were compared: one undergoing electro-acupuncture with TENS (frequency of stimulation was 100 Hz, and the intensity of the electrical stimulus varied from 0.1 to 1.0 mA for 30 min) and two as a control, one with a sham acupuncture point and another from a migraineur wait-list control group in a unique session (Yang et al., 2014). This protocol had more differences in parameters, and compared two groups: one undergoing electro-acupuncture with TENS (wave pulse and a current of 0.5 mA; an output of 6–9 volts would be delivered at 10–20 Hz for 20 min) for 10 sessions, and another with drug therapy (flunarizine 20 mg OD along with tab paracetamol 500 mg SOS), both for 30 days (Vijayalakshmi et al., 2014). Only one differed in days of application at acupuncture points using at-VNS at 1 Hz and 0.2 ms of intensity in the left cymba concha, and a sham at-VNS in 7 days of stimulation of 8 min each. A decrease in migraine pain intensity, but not in the other outcomes, was identified (Luo et al., 2020).

There were four studies where the parameters in their protocols were similar enough to be able to compare the results obtained in each one. One of them used at-VNS, with 1 Hz and 0.2 ms of duration for 30 min each session during 12 sessions in total in the left cymba concha, and a sham at-VNS group where the electrodes were placed in the left tail of the helix. They discovered a positive effect on migraine days, pain intensity, and migraine attack times compared with the sham group, without difference for MSQ, SAS, and SDS (Zhang et al., 2021). The same author published another study with the parameters of 1 Hz, width 0.2 ms, and an intensity of 1.5–3 mA, and a sham at-VNS group (non-stimulation), during 8 min in one session. The study suggests some positive effects on pain and MSQ and a relation between the brain system and the modulation of pain (Zhang et al., 2019). One study compared similar outcomes within three groups: Electro-Acupuncture Group (AG), Sham Acupuncture Group (SAG), and Migraineur Wait-List Control Group (MG). For AG, the acupuncture points were the following: Shaoyang meridians, Luxi (TE19), San Yangluo (TE8), and Xi Yangguan (GB33). They used a TENS device (Electrodes) of Han's acupoint nerve stimulator (HANS; model LH 200A; TENS, Nanjing, China) with the following parameters: frequency of stimulation was 100 Hz and the intensity of the electricity stimulus varied from 0.1 to1.0 mA for 30 min. The SAG was designed to choose non-acupuncture points, and the points were the following: anterior border of the insertion of the deltoid muscle, ST 36, and ulnar side of the arm. There was only one application, and two outcomes were measured, with the VAS and PET-CT being measured pre- and post-intervention. The results obtained were the differences in each group's AG (*P* < 0.05) and SAG (*P* < 0.05), but not between them, and there was no difference in MG (*P* = 0.047). Additionally, the last study (Sacca et al., 2022) used two groups of at-VNS— 1 and 20 Hz, respectively—with two fMRI scans. The outcomes used in this study were the migraine duration (in years), number of migraine attacks in the last four weeks, the pain intensity measure from 0 to 100, Migraine-Specific Quality of Life Questionnaire, and fMRI scan. The results obtained were the following: with 1 Hz at-VNS, there was an increase in NTS (Nucleus of the Solitary Tract)/LC (locus coeruleus)–occipital cortex sFC (static) and a decrease in NTS-thalamus compared with 20 Hz, which was an increase in the LC–anterior cingulate cortex (ACC) both compared at baseline. Moreover, 1 Hz at-VNS greater LC-precuneus and LC–inferior parietal cortex sFC than 20 Hz and 20 Hz stimulation produced an increased LC-ACC and LC–super temporal gyrus/insula sFC in comparison with 1 Hz in static FC. In Dynamic FC 1-Hz, taVNS decreased NTS–postcentral gyrus dFC (less variability), and 20-Hz taVNS decreased dFC (Dynamic FC) of the LC–superior temporal gyrus and the LC–occipital cortex. The conclusion of these results was the relationship between the number of migraine attacks in the past four weeks and the NTS-thalamus sFC during pre-taVNS resting state. As a result, the parameters are vital to obtain an effective treatment for people who suffer from migraine.

One study used drug therapy (Group D) as a comparator group vs. the intervention group who received electro-acupuncture (Group A). Group A had 10 sessions of treatment in a 30-day period, and if there were any attacks, the patient could take a 500 mg tablet of paracetamol. Group D took two tablets—one of flunarizine 20 mg and one of paracetamol 500 mg for 30 days. The outcomes were WHO, QOL, and BREF and were measured pre- and post-treatment. The results showed that patients with migraine headaches had a lower quality of life and higher disability scores, and only two study groups showed a significant change. We could determine that the electro-acupuncture group showed better pain relief than the drug therapy group (P = 0.005–0.000) (Vijayalakshmi et al., 2014).

### Pain intensity, migraine attacks, frequency, and duration

There was no difference in four studies regarding pain intensity with VAS score, the number of migraine attacks, and frequency after the stimulation using at-VNS with 1 Hz (Zhang et al., 2019; Luo et al., 2020; Cao et al., 2021; Sacca et al., 2022).

There were positive findings in two studies regarding migraine days and migraine attacks using at-VNS 1 Hz compared with the sham group, and only one study with positive findings in pain intensity using the VAS score (Yang et al., 2014; Straube et al., 2015; Zhang et al., 2021).

### Imagining condition and analysis

Functional magnetic resonance imaging (fMRI) was applied in all studies to measure neuronal activity and brain structure in migraine patients treated by at-VNS and electro-acupuncture. Six studies evaluated the FC (Zhang et al., 2019, 2021; Luo et al., 2020; Cao et al., 2021; Sacca et al., 2022; Wei et al., 2022), one of which employed the sFC and dFC (Sacca et al., 2022), while the others employed the rsFC (Zhang et al., 2019) and FC (Cao et al., 2021; Zhang et al., 2021; Sacca et al., 2022).

### Brain data image

Brain imaging data were reported in seven studies as shown in Table 2.

There were three studies that employed similar protocols of frequency with 1 Hz in the intervention group, but differed in number of sessions and time of the at-VNS application. As a result, there were some similarities in the stimulated brain areas, but there were also differences, one of which being that the fRMI signal was increased in the bilateral putamen, right caudate, right pallidum/anterior insula, right thalamus, and left frontal operculum, along with a decrease in the bilateral precuneus/posterior cingulate cortex (PCC)/hippocampus/precentral gyrus/medial prefrontal gyrus (mPFC)/anterior cingulate cortex (ACC), bilateral LC, left SN, right RN/PBN, left posterior insula, and bilateral superior/middle frontal gyrus during real at-VNS, compared to the baseline. The comparison between at-VNS and the sham group showed an important deactivation in the at-VNS group at the bilateral LC. The rsFC provided LC rsFC with the right temporoparietal junction, and the left secondary somatosensory cortex (S2) significantly increased compared to the sham group (Zhang et al., 2019). Meanwhile, in another study with a similar protocol of at-VNS with 1 Hz and 8 minutes of intervention, the fRMI scan indicated that FC decreased during at-VNS between the left amygdala and left middle frontal gyrus (MFG), left dorso lateral superior frontal gyrus, right supplementary motor area (SMA), bilateral paracentral lobules, bilateral postcingulum gyrus, and right frontal superior medial gyrus, as did the FC of the right amygdala and left MFG (Luo et al., 2020). Additionally, the last study reported the following related to at-VNS with 1 Hz: the fMRI signal was increased along with the connectivity between the motor-related thalamus subregion and anterior cingulate cortex/medial prefrontal cortex, and there was a decrease in connectivity between the occipital cortex-related thalamus subregion and postcentral gyrus/precuneus (Zhang et al., 2021).

On the other hand, there were two studies that employed two groups using different frequencies, but the last one used two sessions instead of a unique session. The study with one session reported, using an fMRI scan, that an increased signal in the following brain areas produced a significant increasing functional connectivity between the PAG and the bilateral middle cingulate cortex (MCC): right precuneus, left middle frontal gyrus (MFG), and left cuneus with 1 Hz. Additionally, comparing 1 vs. 20 Hz, 1 Hz at-VNS increased PAG connectivity with the MCC, right precuneus/posterior cingulate cortex, left insula, and anterior cingulated cortex (ACC) (Cao et al., 2021). In addition, another study consisting of two sessions reported that in sFC and Fc, comparing 1 Hz and 20 Hz at-VNS showed that 1 Hz at-VNS produced a greater LC–precuneus and LC–inferior parietal cortex sFC than 20 Hz. Additionally, dFC 1-Hz at-VNS decreased NTS–postcentral gyrus dFC (less variability), while 20-Hz at-VNS decreased dFC of the LC–superior temporal gyrus and the LC–occipital cortex (Sacca et al., 2022).

There was only one study which compared healthy people with migraine patients and provided a comparison between electro-acupuncture at GB8 compared with the sham group using an fRMI scan, and there was an increase in FC between the PI (posterior insula) and the left precuneus in the electrical acupuncture group at the baseline, as compared with the sham group and intervention group post-intervention. There was a decreased FC between dAI and the right postcentral gyrus found in the baseline intervention group, compared to the sham group and post-intervention group. Additionally, increased FC between the PI and left precuneus was found in the baseline intervention group, compared to the sham group and post-intervention group. The correlation analysis showed that the FC value of the right postcentral gyrus in the baseline intervention group was negatively correlated with the scores of the Hamilton Rating Scale for Depression and Hamilton Rating Scale for Anxiety. The FC value of the left precuneus in the baseline intervention group was positively correlated with the visual analog scale score (Wei et al., 2022).

A study unique in its protocol employed three groups in order to avoid risk of bias and better mask the true effect of at-VNS than others, and reported the scan imaging with an increase in the PET signal in the middle frontal gyrus, postcentral gyrus, precuneus, parahippocampus, cerebellum, and middle cingulate cortex (MCC), and a decrease in the left hemisphere of the Middle Temporal Cortex (MTC) in the acupuncture group compared with the migraine group. Additionally, an increase in the PCC, insula, inferior temporal gyrus, MTC, superior temporal gyrus, postcentral gyrus, fusiform gyrus, inferior parietal lobe, superior parietal lobe, supramarginal gyrus, middle occipital lobe, angular, and precuneus, and a decrease in cerebellum and parahippocampus vs. the sham group was observed (Yang et al., 2014).

The fMRI Scan outcome provides us with some strong evidence regarding the effectiveness, effect, and relationship between Auricular Vagus Nerve Branches (AVNB); using at-VNS or electro-acupuncture with 1 Hz at the left concha had some positive effects in the Frontal Cortex (FC) of the brain and some other areas, such as the periaqueductal gray (PAG), bilateral MCC, right precuneus/posterior cingulate cortex, left MFG, left cuneus, left insula, and ACC (Cao et al., 2021). The at-VNS at GB8 within 1 Hz could improve the right insula subregions and parietal lobe, specifically the right dAI and right postcentral gyrus, as well as the right PI and left precuneus (Martelletti et al., 2018). Another study, in its protocol, also used at-VNS within 1 Hz at the left cymba concha to provide fMRI evidence of the effect in the occipital thalamic seed and the bilateral PoG (Zhang et al., 2021). The signal in the fMRI FC Improved, left amygdala, left MFG, right SMA, left dorsolateral superior frontal gyrus, bilateral paracentral lobules, bilateral post-cingulum gyrus, right frontal superior medial gyrus, and left FC and right SMA with 1 Hz in 8 min of at-VNS stimulation frequency/time in migraine over 4 weeks (Luo et al., 2020). The last study protocol employing at-VNS with 1 Hz in the left cymba concha had better results in fMRI than sham at-VNS, with a greater deactivation at the bilateral LC, rsFC, right temporoparietal junction, and left secondary somatosensory cortex (Zhang et al., 2019). There was only one protocol with three groups using electro-acupuncture in TE8, TE19, and GB33, which obtained better results compared with the migraine group and sham group. The PET-CT proved that the signal was increased in the middle frontal gyrus, postcentral gyrus, the precuneus, parahippocampus, cerebellum, and middle cingulate cortex (MCC), and decreased in the left hemisphere of the Middle Temporal Cortex (MTC) (Yang et al., 2014).

We divided all therapies/interventions and their efficacy into different groups for each study, as shown in Table 4.

**Table 4:** Summary of the efficacy of the interventions.

**References**	**Intervention(s)**	**Intervention duration (sessions/weeks)**	**Efficacy**
**at-VNS vs. at-VNS (different parameters of frequency)**			
Cao et al. (2021), China	G1; taVNS = 20-Hz. G2; taVNS = 1 Hz continuous wave (width: ~ 0.2 ms), stimulation of 8 min Intensity ~4 mA	Unique session	ta-VNS 1 HZ was superior in terms of the number of migraine attacks and functional brain connectivity.
Wei et al. (2022), China	G1; electrical acupuncture with low-frequency pulse therapy instrument G2; healthy people 2 Hz, 1 mA for 8 min	Unique session	G1 (electrical acupuncture) had some influence in brain connectivity with a therapeutic role.
Sacca et al. (2022), USA	G1; taVNS = 1 Hz. G2; taVNS = 20 Hz 8 minutes, 4 mA,	2 sessions	G1 (1 Hz) improves more than G2 (20 Hz) in migraine attacks. Both improved functional brain connectivity.
Straube et al. (2015), Germany	G1; taVNS 25 Hz, 250 μs, cycle: 30 s on, 30 s off G2; ta-VNS 1 Hz 250 μs, cycle: 30s on, 30 s off	4 h per day over 12 weeks	G2; t-VNS at 1 Hz was safe and effective and after 12 weeks showed a reduction of migraine.
**Real at-VNS vs. sham at-VNS therapies**			
Zhang et al. (2021), China	G1 ta-VNS 1 Hz with the duration of 0.2 ms. Stimulation was continuously applied for 30 min. Intensity 1.5–5 mA G2; Sham group = another location	30 min of 12 treatment sessions in total during the 4-week treatment	G1; relieved the symptoms of headache as well as modulated the thalamocortical circuits in migraine patients
Luo et al. (2020), China	G1; taVNS 1 Hz, 0.2 ms. intensity below the pain threshold (vagal afferent fibers) G2; taVNS Sham. (no vagal afferent fibers)	Unique session of MRI scan, total of 6 20 min fMRI runs and 8 min ta-VNS	G1; FC Improved, left amygdala, left MFG, right SMA, left dorsolateral superior frontal gyrus, bilateral paracentral lobules, bilateral postcingulum gyrus, and right frontal superior medial gyrus. Left FC and right SMA in frequency/time in migraine in 4 weeks.
Zhang et al. (2021), China	G1; taVNS = (frequency: 1 Hz; width: 0.2 ms). Stimulation intensity was adjusted to approximately 1.5–3 mA) G2; Sham group = another location	Unique session of fMRI with at-VNS and sham at-VNS	G1; 1 Hz can significantly modulate activity/connectivity of brain regions and pain modulation system in migraine.
**Electro-acupuncture (auricular branch) vs. another technique**			
Yang et al. (2014), China	G1; AG G2; SAG 100 Hz, for 30 min, 1,0 mA G3; MG	30 min of unique session	Acupuncture stimulation at both sub-specific acupoints evokes central mechanism of acupuncture analgesia by neuroimaging measurement.
Vijayalakshmi et al. (2014), India	G1; Electro acupuncture 10-20 Hz, 0.5 mA; an output of 6-9 volts for 20 min G2; Drug therapy; flunarizine 20 mg OD and tab. paracetamol 500 mg SOS	10 sessions for 30 days	G1 improved in QOL and MIDAS.

VAS, visual analog scale; MSQ, Migraine Specific Quality of Life; SDS, Self-rating Depression Scale; SAS, Self-rating Anxiety Scale; NVS, transcutaneous auricular-Nerve Vagus stimulation; fMRI, functional Magnetic Resonance Image; AG, Electro-acupuncture group; SAG, Sham Acupunture Group; MG, Migraineur Wait-List control Group; WHO QOL BREF.

Levels of Evidence based on the Quality Rating Scheme for Studies and Other Evidence modified from the Oxford Centre for Evidence-Based Medicine for rating of individual studies; available online at https://www.cebm.net/2016/05/ocebm-levels-of-evidence/.RCT.

NR, Not reported; LC, locus coeruleus; rsFC, Resting state functional connectivity; SF, Significance; EA, Electro-acupuncture; PCC, MTC, Middle Temporal Cortex; SMA, supplementary motor area; MFG, middle frontal gyrus; MFC, Middle Frontal Cortex.

The first group was at-VNS vs. another group of at-VNS, but with different parameters to compare which frequency was more effective. Additionally, the efficacy was improved in the at-VNS group to 1 Hz, while the pain of migraine attacks was not; a relationship between brain connectivity and the peripheral branch of the vagus nerve in the auricula with superior areas of the brain was shown, such as MCC, right precuneus, left middle frontal gyrus (MFG), left cuneus, PAG, and AC.

The next group was at-VNS compared with sham group studies, where the sham group was the stimulation or vagal fever of the vagus nerve compared to no stimulation. Additionally, the efficacy that we could extract clearly showed that at-VNS had a greater effect on pain relief and migraine attacks compared with the sham group and the brain activity and connectivity in pain modulation with higher brain areas, such as the left amygdala and left middle frontal gyrus (MFG), left dorso lateral superior frontal gyrus, right supplementary motor area (SMA), bilateral paracentral lobules, bilateral postcingulum gyrus, and right frontal superior medial gyrus, as did the FC of the right amygdala and left MFG.

The last group was at-VNS compared with another treatment or technique, such as drug therapy or manual acupuncture. The results showed that at-VNS was superior compared to drug therapy alone and manual acupuncture or acupuncture in distal points.

### Adverse events

There was only one study of the seven which showed side effects; 7% of patients were reported to have dropped out due to the side effects of t-VNS (Straube et al., 2015). The other six studies reported no adverse events or side effects of neuromodulation (t-VNS, at-VNS, ear-electro-acupuncture) (Vijayalakshmi et al., 2014; Yang et al., 2014; Zhang et al., 2019, 2021; Luo et al., 2020; Cao et al., 2021; Wei et al., 2022) (Table 2).

## Discussion

The aim of this systematic review was to determine the effects of at-VNS for managing chronic migraine-associated symptoms. The results suggest that application of at-VNS may have some positive effects at post-treatment on the frequency and intensity of chronic migraine attacks with 1 Hz of application, as compared with the control group. However, because each study exhibits some differences, more studies are required in order to obtain a good protocol with the exact parameters for finding the best treatment option for these patients.

This study reinforces that our theory is an effective and low-cost treatment option (Platzbecker et al., 2020) compared with pharmaceutical treatments which are available but expensive (Martelletti et al., 2018).

Even the latest publications on acupuncture alone for treating migraine have shown the same results as the current review, thus demonstrating that follow-up for this pathology must be applied effectively to determine the presence of a positive or long-term effect (Naguit et al., 2022). Another finding was the small number of studies of high relevance included in the final screening. Moreover, for at-VNS, we excluded two studies which applied it in the neck area; in the auricula application area, only eight studies were found.

Previous systematic reviews included neuromodulations such as transcranial magnetic stimulation (TMS), non-invasive vagal nerve stimulation (nVNS), non-painful remote electrical stimulation (NRES), and external trigeminal nerve stimulation (e-TNS) (Clark et al., 2022; Naguit et al., 2022), as well as transcranial direct current stimulation (tDCS) (Moisset et al., 2020). All reviews suggest a potential positive but small effect for the treatment of migraine (Martelletti et al., 2018; Cai et al., 2021; Moreno-Ajona et al., 2022). One difference between previous reviews and the current one is that most previous reviews focused on acute migraine, whereas our review focused on chronic migraine (Martelletti et al., 2018). Only one other review also targeted chronic migraine, but used tDCS treatment, which is very different from at-VNS (Cai et al., 2021). The outcomes in two studies were similar in the measurement of pain intensity and number of migraine attacks or duration. As a conclusion for the clinical results, there are many different n-VNS for the treatment of migraine; whether acute or chronic, there are positive effects of the different n-VNS applications which must be taken into consideration to ensure a safe treatment choice (Moisset et al., 2020; Cai et al., 2021; Clark et al., 2022; Naguit et al., 2022).

Some publications have investigated the common pathways of tinnitus and migraine, and the underlying mechanisms as to how n-VNS could work through the vagus nerve for proper management of them. They were focused on applying stimulus to the peripheral nervous system (PNS) through the central nervous system (CNS) to higher brain areas, such as the hippocampus, amygdala, anterior cingulate cortex, and hypothalamus (Moisset et al., 2020; Cai et al., 2021; Clark et al., 2022; Naguit et al., 2022).

Meanwhile, this publication specifically focuses on the auricula area to treat chronic migraine through the vagus nerve. One of the publications on n-VNS referred to neck application, which differs from our work and shows different results. The network could represent a relationship between the peripheral nervous system and the CNS related to the gate control as a primary pain relief, as well as with the calcitonin gene-related peptide (CGRP) and its role in the descending pain modulatory system (DPMS) (Marx et al., 2015; Straube et al., 2015; Zhang et al., 2019, 2021; Luo et al., 2020). Additionally, in some studies, at-VNS had effects on the peripheral nervous system which, through the central nervous system, reached higher brain areas, such as the anterior cingulate cortex (ACC), periaqueductal gray (PAG), prefrontal cortex (PFC), cingulate gyrus, supplementary motor area (SMA), amygdala, and thalamus (Martelletti et al., 2018; Moisset et al., 2020; Cai et al., 2021; Clark et al., 2022; Moreno-Ajona et al., 2022; Naguit et al., 2022). Based on the recent literature and the results of this systemic review, there are no clear mechanisms regarding the role of the n-VNS and at-VNS using acupuncture points to treat chronic migraine and their relationship with the peripheral vagus nerve branches and the ANS. However, there are positive results that encourage more studies with better methodology to be conducted in order to extract strong conclusions to clarify the pathways and the relationship that exists between them.

On the other hand, the results from the studies included in this and another review which used fMRI scanning provide a possible relationship between the at-VNS and positive effects in migraine patients and the brainstem. As a result, it could be linked to an influence in the brainstem, such as in the dorsoposterior insula, low medullary brainstem, medial thalamic, ACC, posterior insula, lower medullary brainstem, and medial thalamic/ACC deactivation with the use of at-VNS/n-VNS. These findings provide a convergence of preliminary evidence supporting the relationship between peripheral areas of the branch of the vagus nerve and superior areas of the brain previously demonstrated (Moulton et al., 2008; Lerman et al., 2019). Neuroanatomical findings support the evidence of the relationship between NTS and the following sites: parabrachial area, locus coeruleus, dorsal raphe, periaqueductal gray, thalamus, amygdala, insula, nucleus accumbens, and bed nucleus of the stria terminalis through the left cymba. Moreover, anti-noception is well referenced with the stimulation of the brain areas such as periaqueductal gray, dorsal raphe, and locus coeruleus, each of which activates descending inhibitory pathways to the spinal cord dorsal horn (Frangos and Komisaruk, 2017), even though the anti-depressive and anti-convulsion have similar effects in some similar areas, such as the amygdala, accumben, and hippocampus, and dorsal raphe and locus coeruleus, respectively. Then, based on our included studies—which show abnormalities of these areas compared to those in healthy humans—there are enough findings that support the relationship between AVNB (ear, concha) with the brainstem (ACC, NTS, LC, etc.) in migrainous and healthy people. However, more clinical trials are required to support the evidence for the best parameters of treatment with at-VNS/n-VNS.

Additionally, there are findings that support the relationship between the superior areas of the brainstem and acupuncture points using fMRI. The areas of the brain which were involved were the following: PAG, ACC, left PCC, insula, limbic/paralimbic, and precuneus (Lerman et al., 2019). Moreover, there is further evidence providing results related to other pathologies, such as low back pain, tinnitus, and the connectivity of the network between the brain and peripheral acupuncture points (Cheng et al., 2020). The areas of stimulation in the brain for low back pain were: PFC (prefrontal cortex), insula, cerebellum, SI (secondary somatosensory cortex), and ACC. For tinnitus, similar areas were involved, such as the right MTG (middle temporal gyrus). As we highlighted previously, there is a relationship between peripheral branch nerves and acupuncture points linked to superior brain areas with a change using at-VNS/n-VNS through fMRI.

### Recommendations and future studies

Future research is required to clarify some important points, not least the effectiveness of the technique. Additionally, for future systematic reviews, studies must be published with high-quality research, such as true randomized controlled trials with adequate control groups, to be able to compare the data derived from them. In terms of quality assessment, they have to follow methodology scales, such as high PEDro or GRADE scores. If they are able to reach those, then future meta-analyses will receive high scores in GRADE as well. Additionally, a recent publication regarding CONSORT included more items, reinforcing the need to improve the research quality of future studies. At the same time, the inclusion criteria should include similar protocols of treatments with parameters such as frequency, intensity, time, number of sessions of treatment, and follow up(s) of the same or similar main outcomes. In conclusion, this systematic review summarizes the main points to improve the quality of future studies with scales and homogeneity in the study design to be able to extract conclusions with similarities and low risk of bias (Butcher et al., 2022).

Additionally, it is important, as a recommendation for future research, that portable devices for at-VNS are promoted. This would be an important advancement in terms of self-treatment at any place and any time for the onset of migraine. It would be really helpful for giving patients who suffer from these sudden attacks the possibility of management. A report was previously published regarding the cost and effectiveness and the role of sequence strategies in migraine attacks. The conclusions were that a portable and effective device could change the quality of life of these patients, providing a low-cost option for most of them in terms of treating the onset of a sudden attack (Mwamburi et al., 2018). If this is compared with traditional methods, the low cost, ease of use, improvement of quality of life and social engagement, and the effectiveness of treatment are the main points underlying the advantages of new models vs. traditional ones. It is well reported that new models, such as NEMOS^®^ or gammacore^®^, are safer and more tolerable than traditional ones, such as with a surgical implant because of AEs (Ben-Menachem et al., 2015; Mwamburi et al., 2018).

### Limitations

We should recognize some limitations of the current review. First, most studies had some concerns regarding their methodological quality (Martelletti et al., 2018), even if the results were slightly positive, and low pain intensity increased the risk of bias and affected quality. For these reasons, we cannot obtain any firm conclusion regarding the effectiveness of at-VNS and ear-electro-acupuncture for managing chronic migraine.

The inconsistency of the follow-up periods and the difference between study protocols, as well as the small number of trials available for systematic review, did not permit us to perform a meta-analysis. Furthermore, the study protocols regarding the same parameters, times of application, measurements, and electro-acupuncture points must be similar, even including the same pathology if there is an acute or chronic migraine, for the same reason previously highlighted for strong conclusions, while also following the guidelines and standards from STRICTA (STandards for Reporting Interventions in Clinical Trials of Acupuncture) (MacPherson et al., 2010).

## Conclusions

The current systematic review found low-quality evidence supporting the idea that at-NVS or ear electro-acupuncture may have some positive effects in the treatment of chronic migraine post-treatment in terms of reducing the frequency and intensity of migraine attacks. Additionally, positive effects were shown in fMRI scans and the relationship between peripheral vagus nerve branches with the superior brainstem. The small number of RCTs and the heterogeneity in the data did not permit us to pool data.

## Author contributions

JM-J contributed to the conception and design of this study, takes responsibility for the integrity of the work as a whole, and resolved any disagreements between both reviewers. CF-d-l-P, JP-G, and FG-E contributed to the interpretation of the data. Article drafts were written by DF-H and JM-J and critically revised by all authors. JP-G and FG-E contributed to realizing the suggestions of the discussion. The literature search was made by DF-H and FG-E. The study selection and the quality of the assessment was carried out by CF-d-l-P and DF-H. The final version of the article was approved by all authors.

## References

[B1] Amiri P. Kazeminasab S. Nejadghaderi S. A. Mohammadinasab R. Pourfathi H. Araj-Khodaei M.. (2022). Migraine: a review on its history, global epidemiology, risk factors, and comorbidities. Front. Neurol. 12, 800605. 10.3389/fneur.2021.800605 35281991 PMC8904749

[B2] Badran B. W. Dowdle L. T. Mithoefer O. J. LaBate N. T. Coatsworth J. Brown J. C.. (2018). Neurophysiologic effects of transcutaneous auricular vagus nerve stimulation (taVNS) via electrical stimulation of the tragus: a concurrent taVNS/fMRI study and review. Brain Stimul. 11, 492–500. 10.1016/j.brs.2017.12.009 29361441 PMC6487660

[B3] Ben-Menachem E. Revesz D. Simon B. J. (2015). Surgically implanted and non-invasive vagus nerve stimulation: a review of efficacy, safety and tolerability. Eur. J. Neurol. 22, 1260–1268. 10.1111/ene.12629 25614179 PMC5024045

[B4] Blech B. Starling A. J. (2020). Noninvasive neuromodulation in migraine. Curr. Pain Headache Rep. 24, 78. 10.1007/s11916-020-00914-3 33326063

[B5] Butcher N. J. Monsour A. Mew E. J. Chan A.-W. Moher D. Mayo-Wilson E.. (2022). Guidelines for reporting outcomes in trial reports: the CONSORT-outcomes 2022 extension. JAMA. 328, 2252–2264. 10.1001/jama.2022.21022 36511921

[B6] Cai G. Xia Z. Charvet L. Xiao F. Datta A. Androulakis X. M. A.. (2021). Systematic review and meta-analysis on the efficacy of repeated transcranial direct current stimulation for migraine. J. Pain Res. 14, 1171–1183. 10.2147/JPR.S295704 33953607 PMC8090858

[B7] Cao J. Zhang Y. Li H. Yan Z. Liu X. Hou X.. (2021). Different modulation effects of 1 hz and 20 hz transcutaneous auricular vagus nerve stimulation on the functional connectivity of the periaqueductal gray in patients with migraine. J. Translational Med. 19, 354. 10.1186/s12967-021-03024-9 34404427 PMC8371886

[B8] Cheng S. Xu G. Zhou J. Qu Y. Li Z. He Z.. (2020). A multimodal meta-analysis of structural and functional changes in the brain of tinnitus. Front. Human Neurosci. 14, 28. 10.3389/fnhum.2020.00028 32161526 PMC7053535

[B9] Clark O. Mahjoub A. Osman N. Surmava A. M. Jan S. Lagman-Bartolome A. M.. (2022). Non-invasive neuromodulation in the acute treatment of migraine: a systematic review and meta-analysis of randomized controlled trials. Neurol Sci. 43, 153–165. 10.1007/s10072-021-05664-7 34698941

[B10] de Tommaso M. Vecchio E. Quitadamo S. G. Coppola G. Di Renzo A. Parisi V.. (2021). Pain-related brain connectivity changes in migraine: a narrative review and proof of concept about possible novel treatments interference. Brain Sci. 11, 234. 10.3390/brainsci11020234 33668449 PMC7917911

[B11] Ellrich J. (2006). Long-term depression of orofacial somatosensory processing. Suppl Clin Neurophysiol. 58, 195–208. 10.1016/S1567-424X(09)70069-8 16623332

[B12] Feng M. Zhang Y. Wen Z. Hou X. Ye Y. Fu C.. (2022). Early fractional amplitude of low frequency fluctuation can predict the efficacy of transcutaneous auricular vagus nerve stimulation treatment for migraine without aura. Front. Mol. Neurosci. 15, 778139. 10.3389/fnmol.2022.778139 35283732 PMC8908103

[B13] Frangos E. Komisaruk B. R. (2017). Access to vagal projections via cutaneous electrical stimulation of the neck: fMRI evidence in healthy humans. Brain Stimulat. 10, 19–27. 10.1016/j.brs.2016.10.008 28104084

[B14] GBD 2016 Headache Collaborators (2018). Global, regional, and national burden of migraine and tension-type headache, 1990–2016: a systematic analysis for the Global Burden of Disease Study 2016. Lancet Neurol. 17, 954–976. 10.1016/S1474-4422(18)30322-3 30353868 PMC6191530

[B15] Grassini S. Nordin S. (2017). Comorbidity in migraine with functional somatic syndromes, psychiatric disorders and inflammatory diseases: a matter of central sensitization? Behav Med. 43, 91–99. 10.1080/08964289.2015.1086721 26431372

[B16] Hamer H. M. Bauer S. (2019). Lessons learned from transcutaneous vagus nerve stimulation (tVNS). Epilepsy Res. 153, 83–84. 10.1016/j.eplepsyres.2019.02.015 30952581

[B17] He W. Wang X. Shi H. Shang H. Li L. Jing X.. (2012). Auricular acupuncture and vagal regulation. Evid Based Complement Alternat Med. 2012, 786839. 10.1155/2012/786839 23304215 PMC3523683

[B18] Henssen D. J. H. A. Derks B. van Doorn M. Verhoogt N. Van Cappellen van Walsum A.-M. Staats P.. (2019b). Vagus nerve stimulation for primary headache disorders: An anatomical review to explain a clinical phenomenon. Cephalalgia. 39, 1180–94. 10.1177/0333102419833076 30786731 PMC6643160

[B19] Henssen D. J. H. A. Derks B. van Doorn M. Verhoogt N. C. Staats P. Vissers K.. (2019a). Visualizing the trigeminovagal complex in the human medulla by combining ex-vivo ultra-high resolution structural MRI and polarized light imaging microscopy. Sci. Rep. 9, 11305. 10.1038/s41598-019-47855-5 31383932 PMC6683146

[B20] Higgins J. P. T. Altman D. G. Gøtzsche P. C. Jüni P. Moher D. Oxman A. D.. (2011). The Cochrane Collaboration' s tool for assessing risk of bias in randomised trials. BMJ. 343, d5928. 10.1136/bmj.d5928 22008217 PMC3196245

[B21] Huang T. Wang S. Kheradmand A. (2020). Vestibular migraine: an update on current understanding and future directions. Cephalalgia. 40, 107–121. 10.1177/0333102419869317 31394919

[B22] Kaniusas E. Kampusch S. Tittgemeyer M. Panetsos F. Gines R. F. Papa M.. (2019). Current directions in the auricular vagus nerve stimulation I – a physiological perspective. Front. Neurosci. 13, 1–23. 10.3389/fnins.2019.00854 31447643 PMC6697069

[B23] Lerman I. Davis B. Huang M. Huang C. Sorkin L. Proudfoot J.. (2019). Noninvasive vagus nerve stimulation alters neural response and physiological autonomic tone to noxious thermal challenge. PloS ONE. 14, e0201212. 10.1371/journal.pone.0201212 30759089 PMC6373934

[B24] Luo W. Zhang Y. Yan Z. Liu X. Hou X. Chen W.. (2020). The instant effects of continuous transcutaneous auricular vagus nerve stimulation at acupoints on the functional connectivity of amygdala in migraine without aura: a preliminary study. Neural Plasticity. 2020, 1–13. 10.1155/2020/8870589 33381165 PMC7759401

[B25] MacPherson H. Altman D. G. Hammerschlag R. Youping L. Taixiang W. White A.. (2010). Revision group. revised standards for reporting interventions in clinical trials of acupuncture (STRICTA): extending the CONSORT statement. PLoS Med. 7, e1000261. 10.1371/journal.pmed.1000261 20543992 PMC2882429

[B26] Maher C. G. Sherrington C. Herbert R. D. Moseley A. M. Elkins M. (2003). Reliability of the PEDro scale for rating quality of randomized controlled trials. Physical Therapy. 83, 713–721. 10.1093/ptj/83.8.713 12882612

[B27] Martelletti P. Barbanti P. Grazzi L. Pierangeli G. Rainero I. Geppetti P.. (2018). Consistent effects of non-invasive vagus nerve stimulation (nVNS) for the acute treatment of migraine: additional findings from the randomized, sham-controlled, double-blind PRESTO trial. J. Headache Pain. 19, 101. 10.1186/s10194-018-0949-9 30382909 PMC6755599

[B28] Marx R. G. Wilson S. M. Swiontkowski M. F. (2015). Updating the assignment of levels of evidence. J. Bone. Joint Surg. Am. 97, 1–2. 10.2106/JBJS.N.01112 25568387

[B29] Moisset X. Pereira B. Ciampi D. Andrade D. Fontaine D. Lantéri-Minet M.. (2020). Neuromodulation techniques for acute and preventive migraine treatment: a systematic review and meta-analysis of randomized controlled trials. J. Headache Pain. 21, 142. 10.1186/s10194-020-01204-4 33302882 PMC7726868

[B30] Möller M. Mehnert J. Schroeder C. F. May A. (2020). Noninvasive vagus nerve stimulation and the trigeminal autonomic reflex: an fMRI study. Neurology. 94, e1085–e1093. 10.1212/WNL.0000000000008865 32029547

[B31] Moreno-Ajona D. Hoffmann J. Akerman S. (2022). Devices for episodic migraine: past, present, and future. Curr. Pain Headache Rep. 26, 259–265. 10.1007/s11916-022-01024-y 35147856 PMC8930505

[B32] Moulton E. A. Burstein R. Tully S. Hargreaves R. Becerra L. Borsook D.. (2008). Interictal dysfunction of a brainstem descending modulatory center in migraine patients. PloS ONE. 3, e3799. 10.1371/journal.pone.0003799 19030105 PMC2582961

[B33] Mwamburi M. Tenaglia A. T. Leibler E. J. (2018). Cost-effectiveness of noninvasive vagus nerve stimulation for acute treatment of episodic migraine and role in treatment sequence strategies. Am. J. Managed Care. 24, S527–S533. 30543270

[B34] Naguit N. Laeeq S. Jakkoju R. Reghefaoui T. Zahoor H. Yook J. H.. (2022). Is acupuncture safe and effective treatment for migraine? a systematic review of randomized controlled trials. Cureus. (2022) 14, e20888. 10.7759/cureus.20888 35145793 PMC8807499

[B35] Page M. J. McKenzie J. E. Bossuyt P. M. Boutron I. Hoffmann T. C. Mulrow C. D.. (2021). The PRISMA 2020 statement: an updated guideline for reporting systematic reviews. BMJ. 372, n71. 10.1136/bmj.n71 33782057 PMC8005924

[B36] Platzbecker K. Timm F. P. Ashina S. Houle T. T. Eikermann M. (2020). Migraine treatment and the risk of postoperative, pain-related hospital readmissions in migraine patients. Cephalalgia. 40, 1622–1632. 10.1177/0333102420949857 32838537

[B37] Rong P. J. Fang J. L. Wang L. P. Meng H. Liu J. Ma Y.. (2012). Transcutaneous vagus nerve stimulation for the treatment of depression: a study protocol for a double blinded randomized clinical trial. BMC Complement. Altern. Med. 12, 255. 10.1186/1472-6882-12-255 23241431 PMC3537743

[B38] Sacca V. Zhang Y. Cao J. Li H. Yan Z. Ye Y.. (2022). evaluation of the modulation effects evoked by different transcutaneous auricular vagus nerve stimulation frequencies along the central vagus nerve pathway in migraines: a functional magnetic resonance imaging study. Neuromodulation. 26, 620–628. 10.1016/j.neurom.2022.08.459 36307355

[B39] Silberstein S. D Yuan H Najib U Ailani J de Morais A. L Mathew P. G. (2020). Non-invasive vagus nerve stimulation for primary headache: a clinical update. Cephalalgia. 40, 1370–1384. 10.1177/0333102420941864 32718243

[B40] Simon B. Blake J. (2017). Mechanism of action of non-invasive cervical vagus nerve stimulation for the treatment of primary headaches. Am J Manag Care. 23, S312–6. 29144716

[B41] Song Y. Li T. Ma C. Liu H. Liang F. Yang Y.. (2022). Comparative efficacy of acupuncture-related therapy for migraine: a systematic review and network meta-analysis. Front. Neurol. 13, 1010410. 10.3389/fneur.2022.1010410 36388203 PMC9643721

[B42] Stovner L. J. Andree C. (2010). Prevalence of headache in Europe: a review for the Eurolight project. J. Headache Pain. 11, 289–299. 10.1007/s10194-010-0217-0 20473702 PMC2917556

[B43] Straube A. Ellrich J. Eren O. Blum B. Ruscheweyh R. (2015). Treatment of chronic migraine with transcutaneous stimulation of the auricular branch of the vagal nerve (Auricular t-VNS): a randomized, monocentric clinical trial. J Headache and Pain. 16, 543. 10.1186/s10194-015-0543-3 26156114 PMC4496420

[B44] Tobaldini E. Toschi-Dias E. Appratto D. Vicenzi M. Sandrone G. Cogliati C.. (2019). Cardiac and peripheral autonomic responses to orthostatic stress during transcutaneous vagus nerve stimulation in healthy subjects. J. Clin. Med. 8, 496. 10.3390/jcm8040496 30979068 PMC6517949

[B45] Usichenko T. Hacker H. Lotze M. (2017). Transcutaneous auricular vagal nerve stimulation (taVNS) might be a mechanism behind the analgesic effects of auricular acupuncture. Brain Stimulation. 10, 1042–1044. 10.1016/j.brs.2017.07.013 28803834

[B46] Vetvik K. G. MacGregor E. A. (2017). Sex differences in the epidemiology, clinical features, and pathophysiology of migraine. Lancet Neurol. 16, 76–87. 10.1016/S1474-4422(16)30293-9 27836433

[B47] Vijayalakshmi I. Shankar N. Saxena A. Bhatia M. S. (2014). Comparison of effectiveness of acupuncture therapy and conventional drug therapy on psychological profile of migraine patients. Indian J Physiol Pharmacol. (2014) 58, 69–76. 25464680

[B48] Wei X. Y. Luo S. L. Chen H. Liu S. S. Gong Z. G. Zhan S. H.. (2022). Functional connectivity changes during migraine treatment with electroacupuncture at Shuaigu (GB8). J. Integrat. Med. 20, 237–243. 10.1016/j.joim.2022.01.009 35219625

[B49] Woldeamanuel Y. W. Cowan R. P. (2017). Migraine affects 1 in 10 people worldwide featuring recent rise: a systematic review and meta-analysis of communitybased studies involving 6 million participants. J. Neurol. Sci. 372, 307–315. 10.1016/j.jns.2016.11.071 28017235

[B50] Yang M. Yang J. Zeng F. Liu P. Lai Z. Deng S.. (2014). Electroacupuncture stimulation at sub-specific acupoint and non-acupoint induced distinct brain glucose metabolism change in migraineurs: a PET-CT study. J. Transl. Med. 12, 351. 10.1186/s12967-014-0351-6 25496446 PMC4279794

[B51] Yap J. Y. Y. Keatch C. Lambert E. Woods W. Stoddart P. R. Kameneva T.. (2020). Critical review of transcutaneous vagus nerve stimulation: challenges for translation to clinical practice. Front. Neurosci. 14, 284. 10.3389/fnins.2020.00284 32410932 PMC7199464

[B52] Zhang Y. Huang Y. Li H. Yan Z. Zhang Y. Liu X.. (2021). Transcutaneous auricular vagus nerve stimulation (TaVNS) for migraine: an FMRI study. Regional Anesthesia Pain Med. 46, 145–150. 10.1136/rapm-2020-102088 33262253

[B53] Zhang Y. Liu J. Li H. Yan Z. Liu X. Cao J.. (2019). Transcutaneous auricular vagus nerve stimulation at 1 Hz modulates locus coeruleus activity and resting state functional connectivity in patients with migraine: an fMRI study. NeuroImage Clin. 24, 1–8. 10.1016/j.nicl.2019.101971 31648171 PMC7239932

